# Signalment, clinicopathological findings, management practices and comorbidities in cats with diabetes mellitus in Germany: cross-sectional study of 144 cases

**DOI:** 10.1177/1098612X241303303

**Published:** 2025-01-07

**Authors:** Bente Guse, Judith Langenstein, Natali Bauer, Katarina Hazuchova

**Affiliations:** 1 Clinic for Small Animals (Internal Medicine, Clinical Pathology and Clinical Pathophysiology), Justus-Liebig-University Giessen, Germany; 2 Antech Lab Germany, Augsburg, Germany

**Keywords:** Cobalamin, vitamin B12, pancreatitis, insulin-like growth factor 1, hypocobalaminaemia, hypersomatotropism, hyperthyroidism

## Abstract

**Objectives:**

The aim of this study was to describe signalment, clinicopathological findings, management practices and the occurrence of comorbidities in feline diabetes mellitus (DM) in Germany.

**Methods:**

This was a cross-sectional study using questionnaires and laboratory submissions to a commercial laboratory, Antech Lab Germany, between May 2021 and July 2022. Inclusion criteria were diagnosis of DM by the attending veterinarian and submission of a completed questionnaire besides blood samples. Laboratory testing included haematology, serum biochemistry, concentration of total thyroxine (TT4), insulin-like growth factor 1 (IGF-1), cobalamin (COB), fructosamine, b-hydroxybutyrate and DGGR (1,2-*O*-dilauryl-*rac*-glycero-3-glutaric acid-[6′-methylresorufin] ester) lipase activity. Data are presented as the median (range) and analysed by non-parametric tests. *P* <0.05 was considered statistically significant.

**Results:**

The median (range) age of the 144 diabetic cats at diagnosis was 11 years (0.9–18.7), 66.4% were male, 84.6% were domestic shorthair, 50.4% were currently overweight and 61.5% were previously overweight (body condition score >5/9). Most cats were treated with insulin (84%), most commonly protamine zinc insulin (57.5%). Blood glucose curves or continuous glucose monitoring alone or in combination with other methods were performed to adjust insulin therapy in 70.6% of cats. Based on questionnaires, 78.6% were poorly controlled and 21.4% were well controlled. Increased TT4 occurred in 3/139 and hyperthyroidism was known in 5/139 cats (frequency of known/suspected hyperthyroidism: 5.8% [n = 8/139]); 17.5% (n = 17/97) had increased IGF-1 (IGF-1 >746 ng/ml, cut-off for hypersomatotropism with the chemiluminescence assay used in this study); 24.5% (n = 34/139) had COB <295.2 pmol/l and 54.2% (n = 78/144) had increased DGGR. Cats with IGF-1 >746 ng/ml were receiving a higher insulin dose than cats with IGF-1 ≤746 ng/ml (median 1.63 vs 0.86 U/kg/day, *P* = 0.018).

**Conclusion and relevance:**

Increased DGGR and increased IGF-1 indicating hypersomatotropism are common in diabetic cats and should be tested for. Almost one-quarter of diabetic cats might require COB supplementation.

## Introduction

Diabetes mellitus (DM) is a common feline endocrinopathy. Based on risk factors such as increasing age,^1–6^ obesity^1,3,7–9^ and physical inactivity,^9–11^ in most cats, the disease resembles human type 2 DM.^12^ A proportion of cats, however, might suffer from other conditions, which in human medicine are classified as ‘specific types of diabetes due to other causes’. In humans, these include diseases of the exocrine pancreas (eg, pancreatitis, pancreatic cancer) or endocrinopathies causing insulin resistance (eg, hypersomatotropism, hyperadrenocorticism),^13^ which have been identified as potential causes of DM in cats too.^14,15^ Project ALIVE (Agreeing Language in Veterinary Endocrinology) recently proposed aetiological classification of DM into two main categories: insulin deficient DM (beta-cell-related disorders) and insulin resistant DM (target-organ disorders). While pancreatitis has been included among the potential causes of insulin deficient DM, several endocrinopathies have been listed as causes of insulin resistant DM.^16^ With pancreatitis, there is still a debate if this is the cause or consequence of DM in cats,^17^ and potentially both scenarios are true in individual cats, similar to diabetic humans.^18,19^ In the context of pancreatitis, especially concerning its chronic form, assessment of cobalamin (COB) concentration might be of interest because intrinsic factor, which is necessary for the absorption of COB from the small intestine, is exclusively produced in the pancreas in cats.^20^ However, to the authors’ knowledge, there are no studies reporting on COB concentrations in diabetic cats.

Unlike pancreatitis, there is little doubt that endocrinopathies causing insulin resistance such as hypersomatotropism and hyperadrenocorticism represent the underlying cause of DM in the affected cats.^14,15^ This is substantiated by the fact that 71–92% of cats with DM and hypersomatotropism enter diabetic remission following hypophysectomy.^21,22^ This is also possible in cats with hyperadrenocorticism following hypophysectomy or medical therapy with trilostane,^23,24^ although the disease is generally rare. Some geographical differences in the prevalence of hypersomatotropism seem to exist, with 24.8%,^25^ 17.8%^26^ and 14.9%^27^ of diabetic cats being affected by this condition in the UK, Netherlands and Switzerland, and Argentina, respectively. Therefore, regional knowledge of not only the frequency of hypersomatotropism, but also other conditions causing or accompanying DM in cats might be useful to help clinicians to choose appropriate tests, especially when financial constraints exist. Because any concurrent conditions might negatively affect diabetic control, their early recognition and appropriate treatment, if possible, is also important to improve outcome. Such data, however, have not yet been collected in Germany.

Besides insights into the frequency of underlying or concurrent diseases, knowledge of local insulin management, dietary and monitoring strategies might be useful not only to veterinarians and researchers, but also other parties involved in veterinary care (eg, insurance companies, pharmaceutical companies) to facilitate disease management in that particular region. Although management guidelines for DM exist,^14,15^ their implementation in veterinary practices is largely unknown. Again, there is no information on managing feline DM in German veterinary practices.

The aims of this study, therefore, were to describe signalment, clinicopathological findings and management of diabetic cats in German veterinary practices, as well as to estimate the occurrence of comorbidities in feline DM in Germany. Additional aims were to assess for associations between increased DGGR (1,2-*O*-dilauryl-*rac*-glycero glutaric acid-(6′-methylresorufin) ester) lipase activity (suggestive of pancreatitis), hypocobalaminaemia and gastrointestinal signs in diabetic cats, as well as to assess whether certain factors (signalment, fructosamine concentration, insulin dose) are associated with increased insulin-like growth factor 1 (IGF-1) concentration (suspicious of hypersomatotropism).

## Materials and methods

### Inclusion criteria and laboratory data

This was a cross-sectional study conducted in cooperation with a large commercial laboratory Antech Lab Germany (formerly SYNLAB.vet) with five locations in Germany between May 2021 and July 2022. Veterinary practices and owners of diabetic cats could participate if they submitted a completed questionnaire (see Appendix 1 in the supplementary material) alongside blood samples for haematology (ADVIA 2120i; Siemens Diagnostics), serum biochemistry, DGGR ester lipase activity, concentration of fructosamine, b-hydroxybutyrate (BHB) (all measured by AU 5800, AU 680; Beckman Coulter and Alinity CI-Systems), total thyroxine (TT4), COB and IGF-1^28^ (all three measured using IMMULITE 2000 XPi Immunoassay System; Siemens Medical Solutions Diagnostics). Inclusion criteria were diagnosis of DM by the attending veterinarian and submission of a completed questionnaire with the blood samples. All blood tests were performed free of charge to the owner. Signalment, DM management, diabetic control, laboratory results, frequencies of certain comorbidities/laboratory abnormalities and the presence of complications of DM were evaluated.

### Questionnaire, signalment and management of feline DM

The questionnaire (see Appendix 1 in the supplementary materials) included information on signalment (including body weight and body condition score [BCS] on a nine-point scale),^29^ DM management (insulin, diet, monitoring), comorbidities, complications, medications other than insulin and presence of gastrointestinal signs. If applicable, multiple answers could be given where appropriate. If multiple submissions from the same cat were received, only the earliest submission was included. Cats with BCS of 1–3, 4–5 and 6–9 were considered to be underweight, have an ideal body weight or be overweight, respectively.

### Diabetic control

Diabetic control was considered good or poor based on the information provided in the questionnaire by the submitting veterinarian. Diabetic control was reported in all cats and in cats treated with insulin for >6 months because, in cats not yet or only recently treated with insulin, the length of treatment might not be sufficient to achieve good diabetic control. In poorly controlled cats, veterinarians were asked to specify the criteria used for this assessment.

### Laboratory results

In addition to concentration of fructosamine, TT4, IGF-1, COB, BHB and DGGR activity, selected haematology and biochemistry parameters were analysed: red blood cell count (RBC), activity of alkaline phosphatase (AP) and alanine aminotransferase (ALT), concentration of creatinine (CREA), bilirubin (BILI), cholesterol (CHOL) and triglycerides (TRI). Note that, for clarity, the above defined abbreviations of laboratory parameters are used throughout the manuscript without the term ‘concentration’ or ‘activity’.

RBC rather than haematocrit or packed cell volume was evaluated because the latter parameters are affected by erythrocyte swelling during storage, which can lead to significantly increased values within 12 h of blood collection.^30^ When reporting TT4 and COB, cats treated with antithyroid drugs and cats receiving COB supplementation (based on information provided in the questionnaire) were excluded, respectively. Because RI for COB was based on the literature and not laboratory-own data, the proportion of cats with COB <295.2 pmol/l necessitating supplementation^31,32^ was further evaluated (see below).

### Comorbidities and laboratory abnormalities suggesting the presence of pancreatitis or need for COB supplementation

Comorbidities could be directly listed by the veterinarian in the questionnaire (eg, heart disease, chronic kidney disease [CKD]) or were identified based on the laboratory tests as a part of this study (suspected hypersomatotropism, suspected uncontrolled hyperthyroidism). Hypersomatotropism was suspected based on IGF-1 >746 ng/ml, indicating hypersomatotropism with a sensitivity of 84.4% and a specificity of 97.2%.^28^ For calculation of the frequency of suspected hypersomatotropism and associated comparisons, cats with untreated diabetes or diabetes treated for less than 1 month were excluded, because the IGF-1 concentration might be low in newly diagnosed diabetic cats and has been shown to increase 2–4 weeks after treatment start.^33^ Uncontrolled hyperthyroidism was suspected based on TT4 above the laboratory reference interval (RI) (TT4 >46.4 nmol/l). Because diagnosis of hyperthyroidism should be made based on combination of corresponding clinical signs and laboratory findings (increased TT4 and/or increased free T4),^34^ the present study reports the frequency of known (where indicated in the questionnaire) and suspected (based on TT4 >RI) hyperthyroidism. Laboratory abnormalities including increased DGGR suggesting the presence of pancreatitis (DGGR ⩾27 U/l),^35,36^ and COB <295.2 pmol/l (corresponding to 400 ng/l, indicating the need for COB supplementation based on the recommendation of Texas A&M)^31,32^ were also analysed but were not counted as ‘comorbidities’ because diagnosis of pancreatitis cannot be made on a single DGGR measurement^37^ and COB <295.2 pmol/l can be caused by various conditions.^20^ Similarly, other laboratory abnormalities such as azotaemia (indicated by CREA >RI or ⩾140 µmol/l based on International Renal Interest Society [IRIS] guidelines)^38^ or anaemia (indicated by an RBC < RI) were not counted as comorbidities because they can be caused by a number of conditions.^39^

Selected parameters were compared between cats with and without suspected hypersomatotropism (sex, age, fructosamine concentration, insulin dose); between cats with and without increased DGGR (occurrence of gastrointestinal signs; proportion of cats with COB <295.2 pmol/l); and between cats with and without the need for COB supplementation (occurrence of gastrointestinal signs). Gastrointestinal signs were defined as vomiting, diarrhoea and inappetence. Since weight loss can also be a clinical sign of DM,^14^ it was not included.

### Complications

Complications of DM such as hypoglycaemia^40^ or diabetic ketoacidosis (DKA)^41^ were also listed by veterinarians in the questionnaire. The proportion of cats with BHB >2.4 mmol/l, indicating DKA with a sensitivity and specificity of 100% and 87%, respectively,^42^ was reported.

### Statistical analysis

Statistical analysis was performed using SPSS, version 29.0.1.1 (IBM). The data were assessed for normality by the Shapiro–Wilk test. Because the majority of the data were non-normally distributed, they are presented as the median (range). Frequencies (known or suspected hyperthyroidism, suspected hypersomatotropism, increased DGGR, COB indicating the need for supplementation)^31^ are reported as the proportion (%) and 95% confidence interval (CI). A Mann–Whitney *U*-test was used to compare age, fructosamine concentration and insulin dose between cats with and without suspected hypersomatotropism. Categorical variables were compared using the χ^2^ test. *P* <0.05 was considered statistically significant.

## Results

### Signalment and management of feline DM

Laboratory data and completed questionnaires from 163 cats were available. After the removal of 19 duplicates, 144 cats remained in the study. Median (range) age at DM diagnosis (n = 132/144) was 11 years (0.9–18.7). Most cats (n = 95/143; 66.4%) were male and domestic shorthair (n = 121/143; 84.6%). Median (range) weight (n = 121/144) was 5.2 kg (2.57–11), with 50.4% (n = 60/119) of cats being overweight at the time of study participation and 61.5% (n = 80/130) having a history of obesity. Only 11.8% (n = 15/127) were treated with steroids within 6 months prior to diagnosis. Further details on signalment are provided in Table 1.

**Table 1:** Breed, sex and body condition score of 144 diabetic cats based on questionnaires submitted alongside blood samples for laboratory tests

Parameter/question	Choice of options	n (%)
Breed (n = 143)	Domestic shorthair	121 (84.6)
	Maine Coon	5 (3.5)
	Norwegian Forest Cat	4 (2.8)
	British Shorthair	4 (2.8)
	Persian	3 (2.1)
	Other	6 (4.2)
Sex (n = 143)	Male castrated	94 (65.7)
	Male intact	1 (0.7)
	Female spayed	47 (32.9)
	Female intact	1 (0.7)
Body condition score (n = 119)	Underweight (1–3)	21 (17.7)
	Normal weight (4–5)	38 (31.9)
	Overweight (6–9)	60 (50.4)

Most cats (n = 42/142; 29.6%) had been treated with insulin for 1–6 months, with 92.6% (n = 112/121) receiving insulin twice daily at a median dose of 0.85 U/kg/day (0.05–3.19; n = 101/121), but 15.3% (n = 22/144) were newly diagnosed and not yet treated with insulin. Protamine zinc insulin was the most commonly used insulin type (n = 69/120; 57.5%); 42.5% (n = 60/141) cats were fed a diabetic prescription diet. Most common monitoring tools were fructosamine measurement (n = 65/126; 51.6%) and blood glucose curves (BGCs) at home (n = 63/126; 50%). Further details on DM management are provided in Table 2. The list of medications other than insulin is provided in Appendix 2 in the supplementary material.

**Table 2:** Questionnaire results regarding management of diabetes mellitus in 144 cats (multiple answers were possible regarding criteria used to adjust the insulin dose, and the frequencies are calculated out of the total number of cats for which this question was answered)

Parameter/question	Choice of options	n (%)
Insulin treatment (n = 144)	Yes	121 (84.0)
	No Not yet because newly diagnosed	1 (0.7) 22 (15.3)
Duration of insulin treatment (n = 142)	Not yet	22 (15.5)
	<1 month	15 (10.6)
	1–6 months	42 (29.6)
	6 months to 1 year	19 (13.4)
	1–2 years	15 (10.5)
	>2 years	29 (20.4)
Insulin preparation (n = 120)	Porcine lente insulin	18 (15.0)
	Protamine zinc insulin	69 (57.5)
	Insulin glargine	31 (25.8)
	Insulin detemir	2 (1.7)
Frequency of insulin administration (n = 121)	Once daily	7 (5.8)
	Twice daily	112 (92.6)
	Other	2 (1.7)
Diabetic diet (n = 141)	Yes	60 (42.5)
	No	81 (57.5)
Food consistency (n = 142)	Dry food	24 (16.9)
	Wet food	37 (26.1)
	Mixed	79 (55.6)
	Other	2 (1.4)
Frequency of feeding (n = 141)	Once daily	0 (0)
	Twice daily	58 (41.1)
	Other	83 (58.9)
Criteria used to adjust insulin dose (n = 126)	Fructosamine	65 (51.6)
	BGC (at veterinary practice)	23 (18.3)
	BGC (at home)	63 (50.0)
	Continuous glucose monitoring	18 (14.3)
	Spot glucose at the time of nadir	28 (22.2)
	Spot glucose before insulin injection	28 (22.2)
	Glucosuria	9 (7.1)

BGC = blood glucose curve

### Diabetic control

Information regarding diabetic control was available in 131/144 cases, where 21.4% (n = 28/131) were well and 78.6% (n = 103/131) were poorly controlled. Considering only cats that had been treated with insulin for >6 months, 28.8% (n = 17/59) were classified as well controlled and 71.2% (n = 42/59) as poorly controlled based on the questionnaire. Polyuria/polydipsia was the most frequently used indicator of poor diabetic control (n = 56/96; 58.3%), followed by weight loss (n = 44/96; 45.8%) (Table 3).

**Table 3:** Indicators of poor diabetic control based on questionnaire results (multiple answers were possible and the frequencies are calculated out of the total number of cats for which this question was answered)

Criteria used to assess clinical diabetic control in 96 cats	
Polyuria/polydipsia	56 (58.3)
Polyphagia	36 (37.5)
Weight loss	44 (45.8)
Increased fructosamines	40 (41.7)
Hyperglycaemia in single glucose measurements	43 (44.8)
Hyperglycaemia in BGC	33 (34.4)
Other	3 (3.1)

Data are n (%)

BGC = blood glucose curve

### Laboratory results

Most cats had increased fructosamine (n = 124/144; 86.1%) and 44.0% (n = 62/141) had elevated BHB. About one-third had elevated liver enzymes (AP 29.1% [n = 41/141], ALT 34.8% [n = 49/141]) and a decreased TT4 (n = 43/134, 32.1%). Anaemia (RBC < RI) was detected in 22.6% (n = 31/137) cats, and azotaemia was present in 14.9% (n = 21/141) based on CREA >RI and in 28.4% (n = 40/141) based on IRIS guidelines (CREA ⩾140 µmol/l) (Table 4).^38^

**Table 4:** Clinicopathological findings in 144 diabetic cats

Parameter (unit)	Total number (n)	Laboratory reference interval	Median (range)	Decreased n (%)	Normal n (%)	Increased n (%)
RBC (10^12^/l)	137	7.2–11.0	8.6 (4.7–12.5)	31 (22.6)	100 (73.0)	6 (4.4)
CREA (µmol/l)	141	60–166	116 (54–419)	1 (0.7)	119 (84.4)	21 (14.9)
AP (U/l)	141	<66	52 (7– 211)	–	100 (70.9)	41 (29.1)
ALT (U/l)	141	<102	77 (29–547)	–	92 (65.2)	49 (34.8)
TT4 (nmol/l)	134	12.2–46.4	15.9 (<6.4–82.8)	43 (32.1)	88 (65.7)	3 (2.2)
BILI (µmol/l)	141	<4.1	3.4 (<1.7–78.7)	–	111 (78.7)	30 (21.3)
CHOL (mmol/l)	141	2.7–9.0	5.6 (2.1–11.6)	2 (1.4)	131 (92.9)	8 (5.7)
TRI (mmol/l)	141	0.3–1.9	0.9 (0.3–18.4)	2 (1.4)	103 (73.1)	36 (25.5)
FRUSA (µmol/l)	144	146–306	491 (199–802)	0 (0)	20 (13.9)	124 (86.1)
IGF-1 (ng/ml)	132	⩽670	384 (<15–2820)	–	106 (80.3)	26 (19.7)
DGGR (U/l)	144	<27	28 (10–434)	–	66 (45.8)	78 (54.2)
COB (pmol/l)	139	664–2066	677.5 (95.2–5252)	67 (48.2)	60 (43.2)	12 (8.6)
BHB (mmol/l)	141	⩽0.2	0.2 (<0.1–9.3)	–	79 (56.0)	62 (44.0)

Cats treated with antithyroid drugs (n = 5) and cats receiving cobalamin supplementation (n = 4) based on information provided in the questionnaire were excluded from the analysis

ALT = alanine aminotransferase activity; AP = alkaline phosphatase activity; BHB = b-hydroxybutyrate concentration; BILI = bilirubin concentration; CHOL = cholesterol concentration; COB = cobalamin concentration; CREA = creatinine concentration; DGGR = 1,2-*O*-dilauryl-*rac*-glycero glutaric acid-(6′-methylresorufin) ester lipase activity; FRUSA = fructosamine concentration; IGF-1 = concentration of insulin-like growth factor 1; RBC = red blood cell count; TRI = triglyceride concentration; TT4 = total thyroxine concentration

### Comorbidities and laboratory abnormalities suggestive of pancreatitis or need for COB supplementation

Comorbidities listed in the questionnaire by submitting veterinarians are shown in Table 5. The most common comorbidity was pancreatitis (n = 19/144; 13.2%). The diseases that were listed as ‘other’ can be found in Appendix 3 in the supplementary material. In 45.1% (n = 65/144) cases, no comorbidities were reported in the questionnaire.

**Table 5:** Comorbidities in 144 diabetic cats listed in questionnaires by submitting veterinarians (multiple answers were possible, and frequencies are calculated out of the total number of cats for which this question was answered)

Parameter/question	Choice of options	n (%)
Concurrent diseases (n = 144)	Pancreatitis	19 (13.2)
	Chronic enteropathy	12 (8.3)
	Cardiac disease	12 (8.3)
	Chronic kidney disease	10 (6.9)
	Hyperthyroidism	5 (3.5)
	Hyperadrenocorticism	0 (0)
	Other	35 (24.3)
	No comorbidities	65 (45.1)

Based on the questionnaire responses, five cats were hyperthyroid and were treated with antithyroid drugs (methimazole [n = 4], carbimazole [n = 1]). In 1/5 cats, the hyperthyroidism was not controlled (TT4 >RI). Additionally, three more cats had TT4 >46.4 nmol/l, raising suspicion of hyperthyroidism, and increasing the frequency of known or suspected hyperthyroidism in this study to 5.8% (95% CI = 2.2–10.1%; n = 8/139).

Hypersomatotropism was not listed in the questionnaire in any cat, but IGF-1 >746 ng/ml was detected in 17/97 (17.5% [95% CI = 11.3–24.7%]), raising suspicion for this condition.^28^ There was no difference in age and fructosamine concentration between cats with suspected hypersomatropism and those with IGF-1 ⩽746 ng/ml (Table 6). Regarding sex, there was a higher frequency of male cats among diabetics with IGF-1 ⩽746 ng/ml (*P* = 0.025). Furthermore, cats with IGF-1 >746 ng/ml received higher insulin doses than cats with IGF-1 ⩽746 ng/ml (*P* = 0.018).

**Table 6:** Comparison of sex, age, fructosamine concentration and insulin dose between diabetic cats with and without increased IGF-1 (IGF-1 ⩽746 ng/ml) suspicious for hypersomatotropism

Comparison between cats with IGF-1 ⩽746 and IGF-1 >746	Total number (n)	IGF-1 ⩽746	IGF-1 >746	*P* value
Male vs female (n [%])	96	58 (72.5) 22 (27.5)	7 (43.8) 9 (56.2)	0.025
Age (years), median (range)	91	10.4 (3.8–18.7)	11.8 (4.7–16.3)	0.38
FRUSA (µmol/l), median (range)	97	486 (216–676)	494 (262–802)	0.37
Insulin dose (U/kg/day), median (range)	81	0.86 (0.05–3.19)	1.63 (0.35–3.00)	0.018

FRUSA = fructosamine concentration; IGF-1 = concentration of insulin-like growth factor 1

Based on questionnaire responses, 19/144 (13.2%) cats suffered from pancreatitis and 14/19 (73.7%) also had DGGR ⩾27 U/l. Increased DGGR ⩾27 U/l suggestive of the presence of pancreatitis was detected in 78/144 (54.2%; 95% CI = 45.8–62.5) cats. Gastrointestinal signs, however, were more prevalent in cats with DGGR <27 U/l (n = 29/44 [65.9%]) than in cats with DGGR ⩾27 U/l (n = 22/51 [43.1%]) (*P* = 0.026). Almost one-third (31.1%, n = 23/74) of cats with DGGR ⩾27 U/l and less than one-fifth (16.9%, n = 11/65) of cats with DGGR <27 U/l had COB <295.2 pmol/l necessitating supplementation,^31^ but this was not significantly different (*P* = 0.053).

Hypocobalaminaemia was not listed in the questionnaire in any cat, but, in four cats (n = 4/144), their veterinarians were providing some form of COB supplementation (it is unknown whether this was based on earlier measurement of low COB). The frequency of COB <295.2 pmol/l^31^ was 24.5% (95% CI = 18.7–30.2, n = 34/139). COB <295.2 pmol/l was not associated with the presence of gastrointestinal signs (16/27 [59.3%] of cats with COB <295.2 pmol/l and 33/66 [50%] of cats with COB ⩾295.2 pmol/l had gastrointestinal signs, *P* = 0.42).

### Complications

Based on questionnaires, 38.9% (n = 56/144) of cats suffered from complications of their DM. The most common complication was clinical hypoglycaemia (n = 25/144; 17.4%), followed by DKA (n = 20/144, 13.9%; four of these cats [20%] had BHB >2.4 mmol/l at the time of study participation) and diabetic neuropathy (n = 16/144; 11.1%); 2.1% (n = 3/144) had a cataract. Based on BHB >2.4 mmol/l, 12/141 (8.5%) of cats were suspicious of DKA at the time of study participation.^42^ All 12 cats with a BHB >2.4 mmol/l were classified as poorly controlled diabetics in the questionnaire and 33.3% (n = 4/12) have not yet been treated with insulin.

## Discussion

This study describes signalment, clinicopathological findings, management and comorbidities in diabetic cats in Germany. Besides increased fructosamine concentration, the most prevalent laboratory abnormality was increased DGGR suggestive of pancreatitis (54.2% cats, n = 78/144). COB concentration <295.2 pmol/l (~400 ng/l) indicating the need for supplementation^31^ was common (24.5%, n = 34/139), as well as IGF-1 >746 ng/ml suggestive of hypersomatotropism (17.5%, n = 17/97).^28^ The frequency of known or suspected hyperthyroidism was 5.8% (n = 8/139). Most cats (63.1%, n = 89/141) suffered from at least one comorbidity (based on questionnaire and IGF-1 and TT4 measurement).

Age (median 11 years, n = 132/144), sex (66.4% male, n = 95/143) and frequency of current (50.4%, n = 60/119) or previous (61.5%, n = 80/130) obesity are in agreement with previous studies where advanced age,^1–6^ male sex^1,3,5,8–10,43^ and obesity^1,3,7–9^ were identified as risk factors for DM in cats. Most diabetics were domestic shorthair, which most likely reflects the cat population in Germany, with most cats being domestic shorthairs. The laboratory abnormalities (mainly increased AP and ALT, increased triglycerides, hyperbilirubinaemia, azotaemia in 14.9–28.4% [n = 21-40/141] of cats) have also been reported in diabetic cats.^43–47^

Almost all cats (99.3%, n = 143/144) were treated or were about to start treatment with insulin (if newly diagnosed). Most cats (57.5%, n = 69/120) were treated with protamine zinc insulin, an insulin with a long duration of action in cats, and were receiving insulin injections twice daily (92.6%, n = 112/121), in accordance with the current DM management guidelines.^14,15^ Fructosamine measurement (51.6%, n = 65/126) and home BGCs (50%, n = 63/126) were the most frequently used DM monitoring methods. BGCs or continuous glucose monitoring (used in 14.3% cats, n = 18/126), alongside clinical signs assessment, are also recommended by current DM management guidelines.^14,15^ On the other hand, single blood glucose measurements or evaluation of glucosuria, which are less suitable for DM monitoring,^14,15^ were used to adjust insulin therapy in 28 (22.2%) and nine (7.1%) cats, respectively.

In 78.6% (n = 103/131) of the cats, the DM was poorly controlled, with polyuria/polydipsia being the main indicator of poor control (58.3% cats, n = 56/96). A possible reason for this finding is that poorly controlled cats were more likely to present to their veterinarian and undergo further testing (including blood tests). Furthermore, in a proportion of cats DM was newly-diagnosed and not yet treated with insulin (n = 20) or treated for <1 month (n = 15), which might be too short to establish good glycaemic control. However, among cats treated with insulin for >6 months, 71.2% (n = 42/59) were also poorly controlled, indicating that other reasons such as comorbidities or management problems might play a role.

Most cats (63.1%, n = 89/141) suffered from one or more comorbidities. Among those listed in the questionnaire, pancreatitis was the most prevalent (13.2%, n = 19/144) and DGGR ⩾27 U/l suggestive of pancreatitis was present in 54.2% (n = 78/144) of cats. Surprisingly, gastrointestinal signs were less common in cats with increased DGGR than in those with normal DGGR (43.1% [n = 22/51] vs 65.9% [n = 29/44]). However, these clinical signs are not pathognomonic and can occur with other diseases (eg, gastroenteropathies, CKD)^48,49^ and cats with pancreatitis also might not show gastrointestinal signs at all.^50,51^ Furthermore, the diagnosis of pancreatitis should be made based on a combination of clinical signs, laboratory findings, imaging studies and, if applicable, cytology/histology rather than a single blood test (DGGR).^37^ Therefore, the frequency of increased DGGR is not equal to prevalence of confirmed pancreatitis but should rather be considered as a possible indicator of the disease.

The frequency of COB concentration <295.2 pmol/l (~400 ng/l) requiring supplementation^31^ was 24.5% (n = 34/139). To the authors’ knowledge, this is the first study to examine COB concentration in diabetic cats. Pancreatitis, particularly its chronic form, might be the underlying mechanism of hypocobalaminaemia in diabetic cats, but other diseases such as chronic enteropathy or exocrine pancreatic insufficiency are also possible.^20^ Because the pancreas is the exclusive source of intrinsic factor in cats,^20^ the connection between hypocobalaminaemia and chronic pancreatitis is well established in this species.^37,52,53^ However, there was no significant difference in the proportion of cats with hypocobalaminaemia between cats with DGGR ⩾27 U/l and those with DGGR within reference interval in our study. Based on the questionnaire, 8.3% (n = 12/144) of the included cats suffered from chronic enteropathy (33.3%, n = 4/12 of these had COB <295.2 pmol/l), while exocrine pancreatic insufficiency was not reported in any of the included cats. Irrespective of the cause, given the high proportion of diabetic cats with a COB concentration <295.2 pmol/l, COB measurement should be included in the workup of a diabetic cat. Future studies should also include assessment of other indicators of COB status such as methylmalonic acid.^54^

In this study cohort, 17.5% (n = 17/97) of cats had increased IGF-1 suspicious for hypersomatotropism. This is lower than previous results from the UK (~25%),^25,55^ but comparable to the Netherlands, Switzerland (17.8%)^26^ and Argentina (14.9%).^27^ Similar to other studies,^25,27^ cats with IGF-1 >746 ng/ml were receiving higher insulin dose than those with IGF-1 <746 ng/ml. However, our study did not identify predisposition of male cats or higher fructosamine concentrations in cats with suspected hypersomatotropism compared with diabetics with IGF-1 <746 ng/ml. Interestingly, the proportion of male cats was higher in cats with IGF-1 <746 ng/ml, although the reason for this finding is unknown. However, it is important to understand that despite the high specificity of IGF-1 >746 ng/ml for hypersomatotropism (97.2%),^28^ repeated sampling and advanced imaging of the head (eg, CT) would be needed to confirm the diagnosis.

The proportion of diabetic cats with known or suspected hyperthyroidism (5.8%, n = 8/139) was slightly higher than in previous studies.^26,27^ However, these studies did not include cats that were already on therapy and had normal TT4. Given the lowering effect of poorly controlled DM on TT4 concentration (non-thyroidal illness),^56^ the proportion of hyperthyroid cats in this and previous studies might be underestimated. The effect of non-thyroidal illness is supported by TT4 < RI in 32.1% (n = 43/134) of included diabetics.

Clinical hypoglycaemia (17.4%, n = 25/144), DKA (13.9%, n = 20/144) and peripheral neuropathy (11.1%, n = 16/144) were the most frequent complications, which are well known to occur in DM.^41,57–59^ Cataract formation is thought to be rare in feline DM,^60^ although some studies suggest a higher prevalence.^61^ In our study, cataract was reported in three cases (2.1%) but whether this was a complication of DM or was caused by other factors, could not be assessed.

The main limitation of the study is that clinical data were obtained using a questionnaire; therefore, some information might have been omitted. Also, poorly controlled diabetic cats or those with comorbidities might be overrepresented because they are more likely to present at their veterinarians and undergo blood testing. Also, this study only can report on frequencies of suspected comorbidities such as hypersomatotropism or laboratory abnormalities such as increased DGGR, because the diagnosis cannot be made based on a single blood test. Furthermore, there were no standardised criteria for assessing diabetic control because one aim of the study was to evaluate which criteria were used by the submitting veterinarians. For this reason, the assessment is subjective and based on various criteria, which might have influenced the proportion of poorly and well controlled diabetics. Finally, adjustments for multiple comparisons were not made in this study due to its exploratory nature;^62^ therefore, any significant results must be interpreted with caution.

## Conclusions

The signalment and clinicopathological findings in German diabetic cats were comparable to previous reports from other countries.^1–6,8–10,43^ The management largely complies with current guidelines,^14,15^ although single glucose measurements and glucosuria were used to adjust insulin dose in some cats. Increased DGGR suggestive of pancreatitis was the most common laboratory abnormality. Comorbidities were seen in >50% of diabetic cats, with hypersomatotropism suspected based on IGF-1 >746 ng/ml in 17.5% (n = 17/97) cats, and known or suspected hyperthyroidism identified in 5.8% (n = 8/139) cats. In 24.5% (n = 34/139) cats, need for COB supplementation was detected.

## Supplemental Material

sj-docx-1-jfm-10.1177_1098612X241303303 – Supplemental material for Signalment, clinicopathological findings, management practices and comorbidities in cats with diabetes mellitus in Germany: cross-sectional study of 144 cases Appendix 1. Questionnaire.

sj-docx-2-jfm-10.1177_1098612X241303303 – Supplemental material for Signalment, clinicopathological findings, management practices and comorbidities in cats with diabetes mellitus in Germany: cross-sectional study of 144 cases Appendix 2. Questionnaire results regarding medications other than insulin.

sj-docx-3-jfm-10.1177_1098612X241303303 – Supplemental material for Signalment, clinicopathological findings, management practices and comorbidities in cats with diabetes mellitus in Germany: cross-sectional study of 144 cases Appendix 3. Questionnaire results regarding concurrent diseases other than listed in the questionnaire.
