# Supplementary material for: Signalment, clinicopathological findings, management practices and comorbidities in cats with diabetes mellitus in Germany: cross-sectional study of 144 cases
Source: J Feline Med Surg. 2025 Jan 7;27(1):1098612X241303303. doi: 10.1177/1098612X241303303 (PMC11707777; doi:10.1177/1098612X241303303)
Supplement: sj-docx-1-jfm-10.1177_1098612X241303303 – Supplemental material for Signalment, clinicopathological findings, management practices and comorbidities in cats with diabetes mellitus in Germany: cross-sectional study of 144 cases [file sj-docx-1-jfm-10.1177_1098612X241303303.docx]

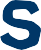

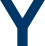

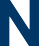

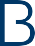

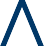


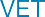


Laboratory -ID:

Date:

Veterinary practice address:

**Questionnaire Diabetes**

| Name of the animal: | | - O Dog ● O Cat |
| --- | --- | --- |
| Age (in years): | Sex:● O male ● O female | Neutered: ●V O yes ● O no |
| Breed: | | Current weight (in kg): |

Current Body Condition Score: 1 to 9 (pictures on a separate sheet)

Was the cat/dog previously overweight? ● O yes ● O no

When was the diabetes diagnosed?

Year Month

Had the cat been treated with steroids within 6 months prior to diagnosis? ● O yes● O no

Is the diabetes treated with insulin? ● O yes O no O not yet, because newly diagnosed

If "no", please state how it is treated:

How long has the dog/cat been treated with insulin?

O not yet O less than 1 month O 1 – 6 months O 6 months – 1 year O 1– 2 years O > 2 years Which insulin preparation is the dog/cat receiving? O Caninsulin O ProZinc O Lantus O other: What is the frequency of insulin administration? ● O once daily O twice daily● O other frequency:

Current insulin dose: IU/cat IU/dog

Is the dog/cat being fed a diabetic diet? ● yy O yes● O no

Feeding: ● O dry food● O wet food● O mixed● O alternative food:

What is the frequency of feeding? O twice daily O other frequency:

Please mark all options that apply to this dog/cat management

What criteria are used to adjust the insulin therapy?

O fructosamine concentration

O blood glucose curves (vet. practice)

O blood glucose curves (at home)

O continuous glucose monitoring

- O spot blood glucose at the time of nadir
- O spot blood glucose before insulin injection
- O glucosuria

Is the dog's/cat's diabetes currently clinically well controlled? ● O yes ● O no

On the basis of which criteria do you consider the diabetes to be not well controlled?

O polyuria/polydipsia

O polyphagia

O weight loss

O increased

fructosamine O hyperglycaemia in spot blood glucose measurements

- O hyperglycaemia in blood glucose curve
- O other:

Were there any complications in the course of the diabetes treatment?

| O diabetic ketosis/ketoacidosis  O clinical hypoglycaemia | - O diabetic cataract - O diabetic neuropathy | - O other: |
| --- | --- | --- |

Does the dog/cat have any other diseases besides diabetes?

| O chronic kidney disease  O pancreatitis  O chronic enteropathy | - O Cushing - O hypothyroidism - O hyperthyroidism | - O heart disease - O other: |
| --- | --- | --- |

Does the dog/cat have symptoms of gastrointestinal disease?

O vomiting ● O diarrhoea● O inappetence ● O weight loss

Is the dog/cat receiving any other medication besides insulin?

O yes ● O no O which one(s)?
