# Supplementary material for: Signalment, clinicopathological findings, management practices and comorbidities in cats with diabetes mellitus in Germany: cross-sectional study of 144 cases
Source: J Feline Med Surg. 2025 Jan 7;27(1):1098612X241303303. doi: 10.1177/1098612X241303303 (PMC11707777; doi:10.1177/1098612X241303303)
Supplement: sj-docx-2-jfm-10.1177_1098612X241303303 – Supplemental material for Signalment, clinicopathological findings, management practices and comorbidities in cats with diabetes mellitus in Germany: cross-sectional study of 144 cases [file sj-docx-2-jfm-10.1177_1098612X241303303.docx]

| **Parameter/Question** | **Choice of options** | **Number n (%)** |
| --- | --- | --- |
| **Medications other than insulin** | yes  no | total: 139  44 (31.7%)  95 (68.3%) |
| **if yes free field to write down which medication** | amlodipine  amoxicillin clavulanic acid  antibiotics (without specification)  atenolol  benazepril  carbimazole  chlorambucil  clopidogrel  enrofloxacin  eye ointment (without specification)  frunevetmab  furosemide  inhalation fluticasone/salmeterol  meloxicam  methimazole  metronidazole  mirtazapine  [oclacitinib](https://www.vetpharm.uzh.ch/Wirkstoffe/000000120831/9269_01.html)  omeprazole  phenobarbital  pimobendan  potassium citrate  prednisolone  propentofyllin  robenacoxib  telmisartan  vitamin B12 | total: 42  1 (2.4%)  2 (4.8%)  1 (2.4%)  1 (2.4%)  1 (2.4%)  1 (2.4%)  1 (2.4%)  1 (2.4%)  1 (2.4%)  1 (2.4%)  1 (2.4%)  2 (4.8%)  1 (2.4%)  7 (16.7%)  4 (9.5%)  1 (2.4%)  2 (4.8%)  1 (2.4%)  1 (2.4%)  1 (2.4%)  2 (4.8%)  1 (2.4%)  3 (7.1%)  1 (2.4%)  1 (2.4%)  1 (2.4%)  4 (9.5%) |
