# Supplementary material for: Signalment, clinicopathological findings, management practices and comorbidities in cats with diabetes mellitus in Germany: cross-sectional study of 144 cases
Source: J Feline Med Surg. 2025 Jan 7;27(1):1098612X241303303. doi: 10.1177/1098612X241303303 (PMC11707777; doi:10.1177/1098612X241303303)
Supplement: sj-docx-3-jfm-10.1177_1098612X241303303 – Supplemental material for Signalment, clinicopathological findings, management practices and comorbidities in cats with diabetes mellitus in Germany: cross-sectional study of 144 cases [file sj-docx-3-jfm-10.1177_1098612X241303303.docx]

| **Other disease** | **Number n (%)** |
| --- | --- |
| arthrosis  atopy  chronic cough  chronic rhinitis  constipation  cystitis  dermatitis  epilepsy  FIV  food allergy  FORL  gingivitis  haematochezia  hepatic lipidosis  hepatic liver enzyme elevation  herpes infection  hypertension  mast cell tumor  *Microsporum canis* infection  otitis  pancreatic carcinoma  spondylosis  urolithiasis | total: 35  8 (22.9%)  1 (2.9%)  1 (2.9%)  2 (5.7%)  1 (2.9%)  1 (2.9%)  4 (11.4%)  1 (2.9%)  1 (2.9%)  1 (2.9%)  2 (5.7%)  1 (2.9%)  1 (2.9%)  1 (2.9%)  1 (2.9%)  1 (2.9%)  1 (2.9%)  1 (2.9%)  1 (2.9%)  1 (2.9%)  1 (2.9%)  1 (2.9%)  2 (5.7%) |
